# A Food, a Bite, a Sip: How Much Allergen Is in That?

**DOI:** 10.3390/nu13020587

**Published:** 2021-02-10

**Authors:** Melanie Kok, Astrid Compagner, Ina Panneman, Aline Sprikkelman, Berber Vlieg-Boerstra

**Affiliations:** 1 Department of Nutrition and Dietetics, Hanze University of Applied Sciences, 9713 GZ Groningen, The Netherlands; melaniekok02@hotmail.com (M.K.); astrid_compagner@hotmail.com (A.C.); 2 The Food College, Practice for Diet, Nutrition, Sports and Food Technology, 8651 AE IJlst, The Netherlands; hetvoedingscollege@online.nl; 3 Department of Pediatric Pulmonology and Allergy, University Medical Center Groningen, University of Groningen, 9700 RB Groningen, The Netherlands; a.b.sprikkelman@umcg.nl; 4 Department of Paediatrics, OLVG Hospital, 1090 HM Amsterdam, The Netherlands

**Keywords:** diet history, food allergy, allergenic protein, thresholds, eliciting dose, bite size, cow’s milk, hen’s egg, peanut, hazelnut

## Abstract

Detailed information about the amount of allergenic protein ingested by the patient prior to an allergic reaction yields valuable information for the diagnosis, guidance and management of food allergy. However, the exact amount of ingredients is often not declared on the label. In this study the feasibility was studied for estimating the amount of allergenic protein from milk, eggs, peanuts and hazelnuts in frequently consumed composite and non-composite foods and per bite or sip size in different age groups in the Netherlands. Foods containing milk, egg, peanut or hazelnut most frequently consumed were selected for the age groups 2–3, 4–6 and 19–30 years. If the label did not yield clear information, the amount of allergenic protein was estimated based on food labels. Bite or sip sizes were determined in these age groups in 30 different foods. The amount of allergenic protein could be estimated in 47/70 (67%) of composite foods, which was complex. Estimated protein content of milk, egg, peanut and hazelnut was 2–3 g for most foods but varied greatly from 3 to 8610 mg and may be below threshold levels of the patient. In contrast, a single bite or sip can contain a sufficient amount of allergenic protein to elicit an allergic reaction. Bite and sip sizes increased with age. In every day practice it is hard to obtain detailed and reliable information about the amount of allergenic protein incorporated in composite foods. We encourage companies to disclose the amount of common allergenic foods on their labels.

## 1. Introduction

For health care professionals who are involved in food allergies, detailed information about the amount of allergenic protein ingested by the patient prior to an allergic reaction yields valuable information for the diagnosis, guidance and management of the food allergy. This information can be obtained by a detailed allergy-focused diet history. One of the aims of the diet history in allergies is to identify suspected foods by linking symptoms to foods [1,2,3,4]. Information about the type and amount ingested which elicited allergic reactions, as well as the severity of the reaction, helps to estimate the clinical sensitivity of the patient and the risk for severe reactions. It is generally accepted that the higher the amount ingested, the more severe the expected allergenic reaction [1,5]. A low eliciting dose is assumed to reflect a higher clinical sensitivity [6] and may therefore be an indication for prescription of an epinephrine auto-injector [7]. This is important information for the design of the oral food challenge test for diagnosis and may lead to more stringent dietary advice. In contrast, in certain patients a high eliciting dose may lead to less stringent dietary advice [8].

The amount of allergenic protein ingested should be estimated by the health care professional from both the portion size ingested and from the amount of allergenic protein present in the food [2,3].

Often the patient has not consumed a full portion of a food but may only have taken one or a few bites or sips from the food until the reaction developed. Thus, in that case the health care professional should estimate the amount of allergenic protein ingested from the size of the bite or sip taken from the food. To our knowledge, no studies have been performed on the bite or sip sizes of foods containing allergenic ingredients.

Secondly, the health care professional should estimate the amount of allergenic protein in the food ingested [2,3]. However, in the majority of composite foods in which the protein content is delivered by several allergenic and non-allergenic ingredients, the exact amount of ingredients is not declared on the label.

The presence of fourteen major food allergens should be fully disclosed on the label in clear wordings according to European regulations. These are milk (including lactose), egg, soy, peanut, tree nuts, gluten, fish, shellfish, mollusks, celery, mustard, lupin, sesame and sulphite [9]. Risk-based approaches to managing allergens in foods are currently being developed by the food industry and regulatory authorities to support food-allergic consumers to avoid ingesting their problem food [10,11]. In non-composite foods or foods having only one protein source, the amount of allergenic ingredients can be derived from the label, e.g., milk contains 3.5% protein from cow’s milk. However, in composite foods most labels do not yield information on the amount of allergenic ingredients unless explicitly stated (e.g., Nutella contains 13% hazelnuts). Thus, most foods lack these data which does not allow the physician or dietitian to accurately estimate the amount of allergenic protein ingested prior to an allergic reaction.

Oral food challenges are the preferred test to establish the diagnosis of food allergy [1,2,4,12]. During oral food challenges, the suspected food is administered to the patient in incrementing amounts with 15–20 minutes time intervals in an open, single-blind or double-blind fashion. Inter-individual thresholds to food allergens widely differ between patients, for reasons not yet fully understood. Patients may react to tiny amounts, such as crumbs of peanut or egg, or to higher doses up to full portions of the allergenic food. Therefore, 6 to 8 dose incremental scales in oral food challenges range from 1 mg protein to more than 4 g protein of the allergenic food, reflecting a full portion size [1,2,12]. Information about the clinical sensitivity of the patient is important for the design of the oral food challenge. Reactions to small amounts in history require increased safety measures during oral food challenges, such as selection of the challenge setting and a lower starting dose [12].

The oral food challenge yields information about the threshold, i.e., the amount of allergenic food eliciting symptoms, as well as the severity of symptoms, although it is recognized that threshold levels in oral food challenges in a clinical setting may be different from threshold levels in everyday life and may not be reproducible [13]. It was recently shown that co-factors such as lack of sleep and physical exercise significantly decrease threshold levels [14].

Once the threshold dose in an oral food challenge is established, insight in the amount of allergenic protein in foods could allow patients with mild symptoms and a high threshold level to expand their diets with foods containing small amounts well below their threshold levels in the absence of known co-factors. However, lack of this information does not allow the dietitian or patient to select foods with allergenic protein below their thresholds to expand the diet of the patient. So far, a more practical approach has been chosen, for example in patients who have passed a baked milk or baked egg challenge. These patients are advised to introduce foods with milk or egg listed as the third ingredient on the label or further down the list [15]. Alternatively, recipes are provided by dietitians to cook or bake their own products with the tolerated amounts of protein incorporated in the recipe [15].

The aims of this study were (1) to study if it is feasible to estimate the amount of allergenic protein from milk, egg, peanut and hazelnut in frequently consumed composite and non-composite foods per portion, per100 g food, and per bite or sip size in different age groups in the Netherlands, and (2) to discuss why it is important to have detailed information of the amount of allergenic protein in foods in the diagnosis and management of food allergies.

## 2. Materials and Methods

### 2.1. The Amount of Allergenic Protein in Foods

**Figure 1:**
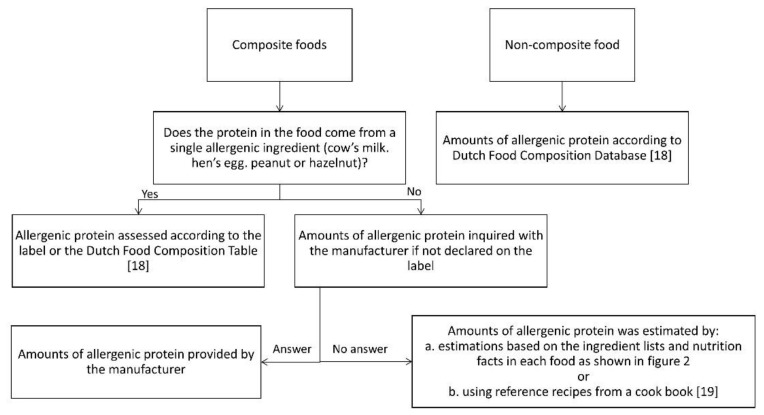
Assessment of the amount of allergenic protein [18,19].

Based on the Dutch National Food Consumption Survey 2011, the most frequently consumed foods were selected for the assessment of the amount of allergenic protein [16,17]. Foods containing milk, egg, peanut or hazelnut consumed by 1% or more of consumers in the age groups 2–3 years, 4–6 years and 19–30 years were selected. Subsequently, the amount of allergenic protein in the selected foods was estimated according to an algorithm (Figure 1), including different methods.

In this study we defined non-composite foods as foods with only one ingredient or having only one ingredient yielding protein. We defined composite foods as foods with multiple ingredients yielding protein.

**Figure 2:**
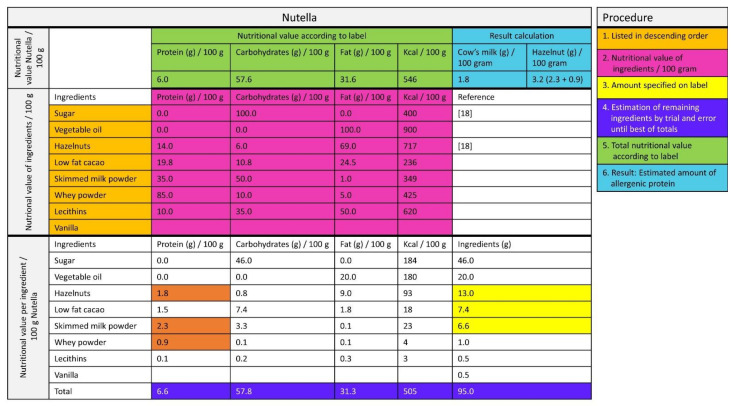
Estimation of the amount of allergen from the label [18].

For non-composite foods, data were derived from the Dutch Food Composition Database 2011 (NEVO) [18] or food labels. For composite foods, the labels were checked for declaration of the amount of allergenic ingredients. If this was not declared, the manufacturer was contacted. When the required information was not provided by the manufacturer, the amount of allergenic protein was, as a non-validated method, estimated by calculations based on the ingredients lists and nutrition facts as follows (Figure 2):

First, it is a given fact that the ingredients on the label are listed in descending order according to their predominance by weight. Second, the nutrition facts (protein, fat, carbohydrates, energy) for each ingredient were relisted per 100 g. Third, the nutrition facts per 100 g were complete when the amount of ingredients was specified on the label (e.g., 13 g of hazelnut, indicated in yellow in Figure 2). Fourth, the amount of the other ingredients was estimated by trial and error until, fifth, the sum of the macronutrients of the ingredients approximated the nutrition facts on the label as closely as possible. Finally, if this method was not feasible, the amount of estimated allergenic protein was based on reference recipes from a Dutch cookbook [19].

Following these assessments, manufacturers were contacted to verify the results of the assessment of the amount of allergenic protein and were requested to comment on our findings.

Results were compared with ED10 and ED50 values for milk, egg, peanut and hazelnut as established in Dutch children and adults by Blom et al. and Klemans et al. [20,21]. ED10 and ED50 is the amount of allergenic protein to which, respectively, 10% and 50% of the allergic subjects react with objective symptoms.

### 2.2. Assessment of Bite and Sip Sizes in Different Age Groups

#### 2.2.1. Selection of Foods

For the three age groups, the top 1% of the most frequently consumed foods containing milk, egg, peanut or hazelnut, as established by the National Food Consumption Survey [16,17], were selected and were allocated into food groups. The four foods most frequently used from each of the food groups were selected for the assessment of bite and sip sizes.

#### 2.2.2. Study Population and Measurements of Bites and Sips

Healthy 2 to 3-year-old children from a preschool, 4 to 6-year-old children from two primary schools, and 19 to 30-year-old students in a nutrition and dietetics faculty were included. Study participants with a food allergy or other conditions that could affect the food intake were excluded.

In the 2–3-year-old children, foods were administered to the children for a bite or sip one by one while playing games. The 4–6-year-old children were asked to take a bite or sip without any instruction and without emphasis on this task to mimic regular bite and sip sizes as closely as possible. Each food was tested in 2–19 individuals in each age group. Each child received a maximum of eight foods. The adults were informed about the purpose of the study and were asked to take a single bite or sip of the food. The adults were asked to test all foods. The food was weighed before and after every bite or sip.

#### 2.2.3. Pilot Study

A pilot study was performed prior to the study at the preschool and in one of the primary schools to test the feasibility of children taking bites or sips. The following essential findings were included in our methods: (1) to keep the attention of the children, all the foods were displayed on site to speed up the process; (2) to ensure a good appetite, the study was performed just before lunch or dinner time; and (3) to imitate the natural meal setting. The study was performed in subgroups of 4–6-year-old children.

#### 2.2.4. Statistics

The results of the study were processed in SPSS. For each food the median intake was calculated in the different age groups and compared using the Mann-Whitney test, as well as the differences in intake between men and women in each age category.

## 3. Results

### 3.1. The Amount of Allergenic Protein in Foods

Ninety-seven foods were selected: 27 non-composite foods for which the amount of allergenic protein was determined using the Dutch NEVO Database [18] or the label (Table 1), and 70 composite foods (Table 2).

For these 70 composite foods, 37 different food manufacturers and two supermarket chains were contacted by telephone and email. Only four different manufacturers provided the required data for four foods.

The amount of allergenic protein of the remaining 66 foods was estimated by the method depicted in Figure 2. The amount of allergenic protein could be estimated in 47/70 (67%) of the composite foods and are listed in Table 2. In 15/47 (32%) of the included composite foods, at least one allergenic ingredient was quantified on the label (e.g., Nutella, 13% hazelnut). For 19/70 (27%) of the composite foods, it was unfeasible to assess the amount of allergenic protein because the nutritional value of the main ingredients could not be estimated. These foods were excluded from further analyses.

Five of the 35 manufacturers responded when verifying these results: three confirmed that the estimated amounts were correct for margarine, filled milk chocolate bar with hazelnuts and hazelnut chocolate bar. Two confirmed that the estimated amounts were incorrect, namely for beef salad and tortellini. According to the manufacturer, beef salad contained 1 g of egg protein per portion instead of 0.5 g according to our estimation. For tortellini, the content of egg protein was 1.19 g per portion instead of 0.91 g per portion. The remaining 30 manufacturers either did not respond or responded but did not confirm or reject the amounts estimated and indicated that they were not willing to share the amount of allergenic protein of their products.

It was found that the actual or estimated amounts of allergenic protein varied widely in foods, and as expected, was highest in non-composite foods (Table 1). Of the non-composite foods with milk, the highest amounts of milk protein per portion were found in skimmed milk 0.1%, semi-skimmed milk 1.5%, whole milk 3.5%, buttermilk, and low-fat Gouda cheese: 9250 mg, 8500 mg, 8250 mg, 7500 mg, and 6840 mg, respectively. The lowest amounts of milk protein per portion were observed for whipping cream, coffee creamer and butter: 230 mg, 50 mg, and 40 mg, respectively. Peanut butter yielded 3200 mg peanut protein per portion.

Of the composite foods with milk (Table 2), the highest amounts of milk protein per portion were found in cheesecake, baby porridge, ice cream, vanilla custard and pancakes: up to 6000 mg, 3840 mg, 3690 mg, 3600 mg, and 1491 mg, respectively. Relatively low amounts of milk protein were found in low-fat margarine, foam sweets banana flavor and filled biscuit, and creamer: 4 mg, 6 mg, 8 mg, and 75 mg of milk protein per portion, respectively.

Of the composite foods with egg (Table 2), the highest amounts of eggprotein per portion were found in pancakes, waffles, ravioli and tortellini: 8610 mg, 1970 mg, 1190 mg and 1190 mg, respectively. Low amounts of egg protein were found in round toast, syrup waffles, penny waffles and Cornetto ice cream: 5 mg, 3 mg, 3 mg, and 3 mg of egg protein per portion, respectively.

Of the composite foods with peanut (Table 2), the amounts of peanut protein per portion varied between 630 mg (peanut cookie) and 2720 mg (coated peanuts).

Of the composite foods with hazelnut (Table 2), the amounts of hazelnut protein per portion varied between 380 mg (Belgium bonbon) and 7 mg (penny waffle).

### 3.2. Comparison of the Estimated Amount of Allergenic Protein to ED10 and ED50

The estimated amount of allergenic milk-, egg-, peanut- and hazelnut-protein per portion were compared to the ED10 and ED50 in children for objective symptoms as established in a Dutch population by Blom et al. [20]. Additionally, the estimated amount of peanut protein per portion was compared to the ED10 and ED50 in children and adults for objective and subjective symptoms by Klemans et al. [21].

- Milk

None of the selected composite or non-composite foods contained less estimated milk protein per portion than the ED10 (4.24 mg), except low-fat margarine. Nine foods contained less estimated milk protein per portion than the ED 50 (156 mg). The other foods contained higher estimated amounts.

- Egg

Four foods contained less estimated egg protein per portion than the ED10 (5.82 mg), while ten foods contained less estimated egg protein per portion than the ED50 (199 mg). All the other foods contained more estimated egg protein per portion.

- Peanut

None of the foods contained less estimated peanut protein per portion than the ED10 (4.42 mg) by Blom [20], the ED10 in children (18.6 mg) and in adults (13.7 mg) by Klemans [21] or the ED50 in children (67,3 mg) by Blom [20]. Only one food contained less peanut protein than the ED50 in adults (821 mg) by Klemans [21].

- Hazelnut

None of the foods contained less estimated hazelnut protein per portion than the ED10 (1.38 mg) by Blom [20]. Two foods contained less estimated hazelnut protein per portion than the ED50 (80.6 mg) by Blom [20].

### 3.3. Assessment of Bite and Sip Sizes in Different Age Groups

Thirty foods were selected: 17 foods for the children 2–3 years of age, 17 foods for the children 4–6 years of age and 19 foods for the adults 19–30 years of age. Several foods were selected for more than one age group. In total, 71 participants were included (41 male (57.7%); 30 females (42.3%)).

In the 2–3-year-old age group, 18 toddlers participated (8 males, 10 females; median 3 years of age). A maximum of eight foods were tested in each child. The sip and bite sizes were close for all foods, except for soft drinks in which the largest median sip size was observed (11 mL) in contrast to milk, in which the smallest median intake was measured (2.5 mL). A large range in bite sizes was measured for pancakes (3.00–9.00 g). There were no significant differences between boys and girls in bite or sip sizes of the selected foods.

In the age group of 4–6-year-old children, 39 children were included (28 males, 11 females; median 4 years). For each child a maximum of eight foods were tested (Table 3). The sip and bite sizes were similar for all foods. Between boys and girls, there was only a significant difference in bite size for pancakes (*p* = 0.008) [19].

In the age group 19–30 years, fourteen adults were included (5 males, 9 females; median age 22 years). There were large differences in the bite and sip sizes for the foods within this age group (Table 3). The largest interquartile range (IQR) was observed for milk (24.00–58.75 mL). Between men and women, significant differences in bite and sip sizes were found for eleven other foods and drinks (*p* values 0.001 to 0.042).

#### 3.3.1. Differences between the Different Age Groups

In the 2–3-year-old children, the bite and sip sizes for wheat bread and mayonnaise were significantly larger than those in 4–6-year-old children (*p* = 0.029 and *p* = 0.012), whereas 4–6-year-old children had significantly larger sip sizes for milk (*p* = 0.010).

For the foods tested in all age groups, the bite and sip sizes of the 19–30-year-old adults were significantly larger compared to the 2–3-year-old children and 4–6-year-old children for all foods.

#### 3.3.2. Amount of Protein per Bite or Sip

In Table 1 and Table 2 it is shown that a single bite or sip of many foods contains sufficient amounts of allergenic protein to elicit an allergic reaction.

## 4. Discussion

This study aimed to test the feasibility of estimating the amount of allergenic protein in frequently consumed foods, as estimated per 100 g, per portion and per bite and sip sizes in different age groups for improved diagnosis and management of food allergies. For non-composite foods, the amount of allergenic protein could easily be derived from the label or food composition tables, as all the protein was delivered by one allergenic ingredient. For composite foods we showed that it is very hard to obtain detailed information about the amount of allergenic protein. Through a lot of effort, the allergenic protein content of many composite foods may at best be estimated, however, true amounts of allergenic protein values may be somehow different.

For most composite foods depicted in Table 2, the amounts of allergenic protein are estimates rather than established amounts of protein. Based on our estimations, most composite foods contain less than 2–3 g of allergenic protein, except a few products that contain higher amounts such as cheesecake, baby porridge, ice cream, vanilla custard and pancakes.

For 19/70 (27%) of the composite foods, the amount of allergenic protein could not be estimated due to lack of detailed information on the label or lack of information from the manufacturer.

Four manufacturers provided us the required protein amounts of four foods (6%). For the other 47/70 (67%) composite foods, the amount of allergenic protein could be estimated using a non-validated method. Only 2/70 (3%) of the composite foods fully disclosed the amount of all allergenic ingredients (Nutella and peanut butter).

If detailed data on the amount of allergenic ingredients were provided by the manufacturer, these data could increase the quality of the diagnosis and management of patients with food allergies. First, full disclosure of not only the presence of allergens but also the amount of allergenic protein on the labels would allow for quantitative risk assessment in diet history and diagnosis. The health care professional could better assess how much allergenic protein is ingested prior to the allergic reaction. These data would help to establish the sensitivity of the patient for the allergenic food in question and, if necessary, sustain decision-making on extra safety measures during oral food challenges in highly sensitive patients. In addition, patients having reacted (severely) to small amounts in history will receive stringent dietary avoidance advice.

Second, detailed data on the amount of allergenic ingredients would support the decision-making for epinephrine auto-injector prescription in clinically sensitive patients.

Third, detailed data on the amount of allergenic ingredients would enable individually tailored dietary advice in food allergic patients. It would allow patients who had a mild reaction to try higher doses in oral food challenge tests to safely introduce foods with small amounts into their diet well below their thresholds. This could include the use of foods containing precautionary labeling, such as “may contain traces of…” [8] or foods containing small amounts of an allergen listed in the ingredient list. This information would allow the dietitian or patient to select foods with allergenic protein below their thresholds to expand the diet. Patients tolerating baked milk and baked egg could introduce products with baked egg and milk into their diets. Lastly, milk and egg ladders, practical tools developed by dietitians to introduce foods at home [22], could be adapted based on the amount of allergen listed on the label.

However, due to a lack of detailed information on the label, the estimations in this study on the amount of allergenic protein do not allow for detailed advice in everyday clinical practice. We therefore encourage companies to disclose the amount of common allergenic foods on their labels.

We do not expect that improved quantitative risk assessment in dietary history will precisely predict the threshold dose during an oral challenge, as exposure in daily life occurs in uncontrolled conditions. Previous studies have shown a lack of correlation between the severity of reactions at home and thresholds or severity during oral food challenges [6,13,14]. This may be due to an incomplete diet history with a lack of data on the exact amount of ingested allergenic food, because thresholds in oral food challenges vary over time and because of co-factors such as sleep deprivation and physical exercise [13,14].

For clinical relevance we compared the amount of estimated allergenic protein per portion with the ED10 and ED50 for allergens as established by several authors [20,21] in the Dutch population. For milk, only one food contained less estimated allergenic protein per portion when comparing the amount of milk in foods to the ED10 for milk; for egg this was found for four foods, while for peanut and hazelnut none of the foods contained less than the ED10 [20,21]. This means that, theoretically, all the other foods will provoke allergic reactions in allergic patients who belong to the 10% most clinically sensitive individuals.

When comparing allergenic protein contents with the ED50, nine foods containing milk, ten foods containing egg, no foods containing peanut and two foods containing hazelnut had allergenic amounts per portion below the ED50 in children. Thus, when taking a diet history, inconsistent reactions may be explained by low amounts of allergenic protein in food, except for peanut. This is even more true when only one of a few bites or sips are taken from the food instead of a full portion.

This study showed a clear difference in bite or sip sizes between the different age groups. As expected, the median bite size increases with age. This difference was significant when comparing the adults with the two younger age groups. We also observed 19–30-year-old men having a larger bite and sip sizes for all types of food than women. We showed that a single bite or sip of many foods contain sufficient amounts of allergenic protein to elicit an allergic reaction.

In the literature, there are some data available about bite and sip sizes, however most studies are performed in adults and in obese versus lean study participants to study the effects of portion size and hunger or satiety on bite or sip sizes [23,24,25,26]. Bite sizes increase with increasing portion size [23,25] and body mass index [24,26]. In our study, regular portions were administered and none of the study participants were extremely obese. Bite and sip size in men were larger than in women [23,24,25], as was found in our study. Our data on bite and sip sizes in both children and adults may further enhance the assessment of the intake of allergenic protein consumed.

Our study has several limitations. We used a non-validated method to assess the amounts of allergenic protein in composite foods. We are not aware of a validated approach, and quantitative measurement of allergenic protein in foods was beyond the scope of this study. We also did not use a power analysis to determine the number of study participants for bite and sip sizes. Therefore, the study participants we used to study bite and sip sizes may not be representative for the different age groups. Bite and sip sizes should therefore be interpreted with caution.

## 5. Conclusions

In conclusion, in everyday practice it is hard to obtain detailed and reliable information about the amount of allergenic protein incorporated in composite foods. Yet, this study provides some insight into the estimated amount of allergenic protein in a large number of commonly consumed foods per portion, per 100 g and also per bite or sip size in the Netherlands, as established using a non-validated method. Diet history may be inconsistent in less sensitive patients as they may not react to foods containing low amounts of allergenic protein. In contrast, a single bite or sip can contain sufficient amount of an allergenic protein to elicit an allergic reaction. Bite and sip sizes increased with age. Disclosure of the amount of allergenic protein on labels would improve quantitative risk assessment in diet history in clinical practice, as well as dietary management of food allergies by allowing patients to introduce foods into their diet that they tolerate.

## Figures and Tables

**Table 1:** Amount of estimated allergenic protein in most frequently consumed non-composite foods in mg or ml per portion, mg or ml per 100 g and mg or ml per median bite or sip size in different age groups.

Food	Composite or Non-Composite Food	Amount of Protein (mg or ml/Portion)	Amount of Protein (mg or ml)/100g)	Amount of Protein (mg or ml)/Median Bite or Sip Size 2–3 Years	Amount of Protein (mg or ml)/Median Bite or Sip Size 4–6 years	Amount of Protein (mg or ml)/Median Bite or Sip Size 19–30 years)
**COW’S MILK**						
**Cheese**						
Cottage cheese [18]	Non-composite food	1680	11,200	100	100	290
Goat cheese fresh [18]	Non-composite food	2010	13,400	120	120	350
Cheese spread 20+ [18]	Non-composite food	2550	17,000	150	150	440
Brie 60+ [18]	Non-composite food	3400	17,000	Nd	Nd	Nd
Goat cheese hard [18]	Non-composite food	4480	22,400	250	250	760
Gouda cheese 48+ [18]	Non-composite food	4560	22,800	250	250	780
Gouda cheese 20+ (low-fat) [18]	Non-composite food	6840	34,200	380	380	1160
**Milk, Milk Products, Milk Replacers and Ice Cream**						
Coffee creamer, powder, low-fat [18]	Non-composite food	50	2000	Nd	Nd	Nd
Coffee creamer full fat [18]	Non-composite food	50	8100	Nd	Nd	Nd
Whipping cream [18]	Non-composite food	230	2300	Nd	Nd	Nd
Crème fraiche [18]	Non-composite food	330	2200	Nd	Nd	Nd
Crème fraiche demi [18]	Non-composite food	450	3000	Nd	Nd	Nd
Sour cream [18]	Non-composite food	470	3100	Nd	Nd	Nd
Fromage frais full fat 8.2% [18]	Non-composite food	1420	7100	Nd	Nd	Nd
Fromage frais low-fat 0.5% [18]	Non-composite food	2020	10,100	610	610	1370
Fromage frais half fat 4.6% [18]	Non-composite food	2300	11,500	690	690	1560
Nutrilon 2 Infant Formula (Nutricia)	Non-composite food	2800	1400	Nd	Nd	Nd
3.5% Full fat yoghurt [18]	Non-composite food	5550	3700	220	220	500
Yoghurt, low-fat 0.3% [18]	Non-composite food	6150	4100	250	250	560
Yoghurt, low-fat 1.5% [18]	Non-composite food	6750	4500	270	270	610
Buttermilk [18]	Non-composite food	7500	3000	Nd	Nd	Nd
Whole Milk 3.5% [18]	Non-composite food	8250	3300	83	264	1056
Semi skimmed milk 1.5% [18]	Non-composite food	8500	3400	85	274	1088
Skimmed milk 0.1% [18]	Non-composite food	9250	3700	93	296	1184
**Fat, Oil and Sauce**						
Butter, salted [18]	Non-composite food	40	700	<10	<10	<10
**HEN’S EGG**						
**Egg**						
Boiled egg [18]	Non-composite food	6200	12,300	308	615	1476
**PEANUT**						
**Spread**						
Peanut butter (Calvé)	Non-composite food	3200	21,420	210	190	560

mg, milligram; ml, milliliter; g, gram; Nd, no data.

**Table 2:** Amount of estimated allergenic protein in most frequently consumed composite foods in mg or ml per portion, mg or ml per 100 g and mg or ml per median bite or sip size in different age groups.

Food	Composite or Non-Composite Food	Amount of Protein (mg or ml/Portion)	Amount of Protein (mg or ml)/100g)	Amount of Protein (mg or ml)/Median Bite or Sip Size 2–3 Years	Amount of Protein (mg or ml)/Median Bite or Sip Size 4–6 years	Amount of Protein (mg or ml)/Median Bite or Sip Size 19–30 years)
**COW’S MILK**						
**Bread and Crackers**						
Currant bread **(Jumbo)	Composite food	590	1700	70	70	100
White bread (Jumbo *)	Composite food	770	1700	30	30	100
**Spread**						
Chocolate hazelnut spread (Nutella)	Composite food	470	3160	30	30	80
**Cake and Biscuits**						
Filled Biscuit (Biscuit fourré **) (Jumbo *)	Composite food	8.75	175	<10	<10	<10
Syrup waffle (Jumbo *)	Composite food	70	180	<10	<10	<10
Waffle (Jumbo *)	Composite food	90	180	<10	<10	20
Penny waffle (Jumbo *)	Composite food	117	780	<10	<10	20
Eclair with whipped cream filling (Roomsoesje **) (Jumbo *)	Composite food	190	1550	Nd	Nd	Nd
Apple flan and crumble topping (Jumbo *)	Composite food	430	430	Nd	Nd	Nd
Cake [19]	Composite food	550	1830	70	70	160
Cheesecake with fromage frais (Dr. Oetker *)	Composite food	2000–6000	2000–6000	Nd	Nd	Nd
**Vegetables**						
Creamed spinach frozen (Iglo)	Composite food	600	1250	50	250	130
**Milk, Milk Products, Milk Replacers and Ice Cream**						
Ice cream dairy, Cornetto Classic (Ola)	Composite food	882	1470	Nd	Nd	Nd
Vanilla custard full fat (Friesland Campina *)	Composite food	3600	2400	140	140	460
Ice cream dairy, cream based (Hertog)	Composite food	3690	2460	Nd	Nd	Nd
Baby Porridge vanilla (Pyjama–papje **) (Nestlé)	Composite food	3840	1920	Nd	Nd	Nd
**Composite Meals**						
Infant jarred food: Lasagna with vegetables (Olvarit)	Composite food	1100	550	Nd	Nd	Nd
Pancakes) [19]	Composite food	1491	2130	90	60	Nd
**Soup**						
Chinese Tomato soup, canned (Unox)	Composite food	110	40	Nd	Nd	<10
**Sweets and Chocolate**						
Foam sweets banana flavor (Bananen schuimpjes **) (Haribo)	Composite food	6	120*	Nd	Nd	Nd
Fudge Caramel Vanilla (Lonka)	Composite food	80	1575	Nd	Nd	Nd
Chocolate bar with hazelnuts (Verkade)	Composite food	180	3510	100	70	210
Filled milk chocolate bar with hazelnuts (BonBon Bloc Praliné milk **) (Cote d’Or)	Composite food	650	4310	130	090	260
Belgium chocolate (Zeevruchten bonbon **) (Isaura)	Composite food	650	4320	130	90	260
**Fat, Oil and Sauce**						
Low-fat margarine (Gouda’s Glorie *)	Composite food	4	80*	<10	<10	<10
Tzatziki (Remia)	Composite food	110	740	Nd	Nd	30
Gravy, powdered (Knorr)	Composite food	110	700	Nd	Nd	Nd
Bechamel sauce [19]	Composite food	1300	8680	Nd	Nd	Nd
**Meat and Poultry**						
Hamburger (Mora)	Composite food	780	1060	20	20	100
Ragout, beef, canned (Unox)	Composite food	175	350	Nd	Nd	Nd
**HEN’S EGG**						
**Bread and Crackers**						
Round toast (Bolletje *)	Composite food	5	50	Nd	Nd	Nd
Round toast, whole wheat (Bolletje *)	Composite food	30	300	Nd	Nd	Nd
**Cake and Biscuits**						
Syrup waffle (Jumbo *)	Composite food	3	6	<10	<10	<10
Penny waffle (Jumbo *)	Composite food	3	33	<10	<10	Nd
Marzipan and chocolate cake (Mergpijpje **) (Jumbo *)	Composite food	100	980	Nd	Nd	Nd
Chocolate coated marsh mellow (Schuimzoenen **) (Buys)	Composite food	140	1400	Nd	Nd	Nd
Eclair with whipped cream filling (Roomsoesje **) (Jumbo *)	Composite food	220	1720	Nd	Nd	Nd
Cake [19]	Composite food	390	1300	50	50	120
Dutch sponge cake (Eierkoek **) (AH)	Composite food	660	2210	90	90	200
Waffle (Jumbo *)	Composite food	1970	3940	118	158	355
**Pasta**						
Ravioli (Grand Italia)	Composite food	1190	2380	Nd	Nd	Nd
Tortellini (Grand Italia)	Composite food	1190	2380	Nd	Nd	Nd
**Milk, Milk Products, Milk Replacers and Ice Cream**						
Ice cream dairy, Cornetto Classic (Ola)	Composite food	3	5	Nd	Nd	Nd
**Snacks, Meals**						
Beef salad (Johma)	Composite food	50	30	Nd	Nd	Nd
**Composite Dishes**						
Egg roll, chicken and ham (Mora)	Composite food	1100	630	Nd	Nd	Nd
Pancakes [19]	Composite food	8610	12,300	492	369	Nd
**Fat, Oil and Sauce**						
Salad cream 25% oil (Slasaus **) (Remia)	Composite food	20	150	Nd	Nd	Nd
Sauce for chips 35% oil (Fritessaus **) (Remia)	Composite food	50	334	<10	20	10
Mayonnaise (Remia)	Composite food	130	840	<10	500	30
**PEANUT**						
**Cake and Biscuits**						
Peanut cookie (Jumbo *)	Composite food	630	6300	63	63	Nd
**Snack Food**						
Japanese rice cracker mix with peanuts (Davis)	Composite food	600	3020	Nd	Nd	Nd
Coated peanuts (Duyvis)	Composite food	2720	13,610	Nd	Nd	Nd
**Sweets and Chocolate**						
Candy bar, Snickers	Composite food	1210	6050	180	180	Nd
M&M’s, chocolate with peanut	Composite food	1160	5800	Nd	Nd	Nd
Peanuts coated with milk chocolate (Chocopinda’s **) (Jumbo *)	Composite food	1260	6300	Nd	Nd	Nd
**Fats, Oils and Savory Sauces**						
Peanut sauce (Wijko)	Composite food	1820	12,100	120	610	420
**HAZELNUT**						
**Spread**						
Chocolate hazelnut spread (Nutella)	Composite food	270	1820	20	20	50
**Cake and Biscuits**						
Penny waffle (Jumbo *)	Composite food	7	70	<10	<10	Nd
**Cereals**						
Muesli (Jumbo *)	Composite food	60	140	Nd	Nd	<10
**Milk, Milk Products, Milk Replacers and Ice Cream**						
Ice cream dairy, Cornetto Classic (Ola)	Composite food	168	280	Nd	Nd	Nd
**Sugar, Sweets, Chocolate and Sweet Sauces**						
Belgium chocolate (Zeevruchten bonbon **) (Isaura)	Composite food	380	2520	80	580	Nd

* Amounts of protein (Dutch Food Composition Database 2011 (NEVO, 2011)) [18] per bite are derived from the bite sizes of wheat bread and reference portion sizes for spread, such as Nutella and peanut butter; ** Amounts of protein (NEVO, 2011) [18] are derived from a comparable food in the NEVO table; mg, milligram; ml, milliliter; g, gram; Nd, no data.

**Table 3:** Median (IQR) bite and sip sizes in different age groups in grams or milliliters.

Type of Food	Median Weight or Volume in Gram or ml * (IQR) *	Number of Participants
**2–3 years of age**		
Wheat bread	2.00 (2.00–3.00)	9
Chocolate hazelnut spread, Nutella *	0.86	n.d.
Peanut butter *	0.86	n.d.
Low-fat margarine *	0.29	n.d.
Cheese *	1.14	n.d.
Milk **	2.50 (2.00–4.25)	6
Boiled egg	2.50 (2.00–4.50)	4
Biscuit (Maria biscuit)	1.00 (1.00–1.00)	8
Crisps (Hamka’s)	0.50 (0.25–0.60)	5
Currant bread	3.00 (1.25–5.50)	4
Pancake	4.00 (3.00–9.00)	5
Snickers	2.50 (1.88–3.25)	6
Soft drink (Taksi) **	11.00 (8.00–12.25)	6
Vanilla custard	6.00 (−)	3
Cake batter	2.00 (1.00–3.50)	5
Fried egg	3.00 (1.00–3.00)	6
Creamed spinach	4.00 (−)	2
Chicken nuggets	2.00 (1.75–3.50)	6
Mayonnaise	1.00 (1.00–2.00)	6
Milk chocolate	2.50 (1.00–4.00)	10
Muffin	3.00 (1.00–4.50)	9
**4–6 years of age**		
Wheat bread	2.00 (1.00–2.00)	17
Hazelnut spread *	0.86	n.d.
Peanut butter *	0.86	n.d.
Low-fat margarine *	0.29	n.d.
Cheese *	1.14	n.d.
Milk **	8.00 (4.00–18.00)	11
Boiled egg	5.00 (3.00–7.75)	16
Biscuit	1.00 (1.00–2.00)	19
Crisps (Hamka’s chips)	0.25 (0.1875–0.5425)	18
Currant bread	3.00 (2.00–4.00)	15
Pancake	3.00 (2.00–5.25)	18
Snickers	3.00 (2.00–4.00)	16
Soft drink (Taksi)	8.00 (4.00–12.00)	17
Vanilla custard	6.00 (3.00–8.50)	13
Cake batter	1.00 (0.50–1.00)	7
Fried egg	3.00 (2.00–4.00)	11
Cream spinach	5.00 (4.00–7.00)	7
Chicken nuggets	2.00 (1.50–4.00)	9
Mayonnaise	0.50 (0.50–1.00)	9
Milk chocolate	2.00 (2.00–3.00)	17
Muffin	4.00 (2.00–6.25)	14
**19–30 years**		
Milk **	32.00 (24.00–58.75)	14
Hardboiled egg	12.00 (7.75–16.50)	14
Crisps (Hamka’s chips)	2.00 (1.00–2.00)	11
Soft drink (Taksi)	36.00 (30.70–43.75)	14
Fried egg	6.50 (5.00–9.25)	14
Cream spinach	10.00 (8.50–13.00)	14
Muffin	9.00 (6.00–11.25)	14
Soft drink (Rivella)	31.50 (27.25–50.75)	14
Canned Soup	9.00 (8.00–10.00)	12
Cappuccino	23.50 (14.50–37.50)	14
White bread	6.00 (3.75–8.25)	14
Hazelnut spread *	2.57	n.d.
Peanut butter *	2.57	n.d.
Low-fat margarine *	0.86	n.d.
Cheese *	3.43	n.d.
Yogurt with muesli (Cruesli)	19.00 (14.00–21.25)	14
Potato croquette	5.50 (4.75–8.25)	14
Schnitzel	9.00 (8.50–10.00)	14
Sate sauce	3.50 (2.00–6.00)	14
Nougat	5.50 (4.75–7.25)	14
Belgium chocolate	6.00 (3.75–10.00)	14
Spiced biscuit	3.00 (3.00–5.00)	14

n.d.: not done. * Amounts of protein (NEVO, 2011) (12) are derived from the bite sizes of wheat bread and reference portion sizes for spreads, such as Nutella and peanut butter (17) ** 1 g is considered equivalent to 1 milliliter.

## Data Availability

The data presented in this study are available on request from the corresponding author. A data sharing agreement will be requested.
